# Identification of Cellular Genes Targeted by KSHV-Encoded MicroRNAs

**DOI:** 10.1371/journal.ppat.0030065

**Published:** 2007-05-11

**Authors:** Mark A Samols, Rebecca L Skalsky, Ann M Maldonado, Alberto Riva, M. Cecilia Lopez, Henry V Baker, Rolf Renne

**Affiliations:** 1 Department of Molecular Genetics and Microbiology, University of Florida College of Medicine, Gainesville, Florida, United States of America; 2 University of Florida Shands Cancer Center, Gainesville, Florida, United States of America; 3 Medical Scientist Training Program, Case Western Reserve University, Cleveland, Ohio, United States of America; 4 Genetics Institute, Gainesville, Florida, United States of America; 5 Department of Surgery, University of Florida College of Medicine, Gainesville, Florida, United States of America; Oregon Health and Science University, United States of America

## Abstract

MicroRNAs (miRNAs) are 19 to 23 nucleotide–long RNAs that post-transcriptionally regulate gene expression. Human cells express several hundred miRNAs which regulate important biological pathways such as development, proliferation, and apoptosis. Recently, 12 miRNA genes have been identified within the genome of Kaposi sarcoma–associated herpesvirus; however, their functions are still unknown. To identify host cellular genes that may be targeted by these novel viral regulators, we performed gene expression profiling in cells stably expressing KSHV-encoded miRNAs. Data analysis revealed a set of 81 genes whose expression was significantly changed in the presence of miRNAs. While the majority of changes were below 2-fold, eight genes were down-regulated between 4- and 20-fold. We confirmed miRNA-dependent regulation for three of these genes and found that protein levels of thrombospondin 1 (THBS1) were decreased >10-fold. THBS1 has previously been reported to be down-regulated in Kaposi sarcoma lesions and has known activity as a strong tumor suppressor and anti-angiogenic factor, exerting its anti-angiogenic effect in part by activating the latent form of TGF-β. We show that reduced THBS1 expression in the presence of viral miRNAs translates into decreased TGF-β activity. These data suggest that KSHV-encoded miRNAs may contribute directly to pathogenesis by down-regulation of THBS1, a major regulator of cell adhesion, migration, and angiogenesis.

## Author Summary

Kaposi sarcoma–associated herpesvirus (KSHV) is a gamma-herpesvirus associated with Kaposi sarcoma, primary effusion lymphoma, and a subset of muticentric Castleman disease. Recently, it was found that KSHV encodes 12 microRNAs (miRNAs) within its latency-associated region. miRNAs are small ∼22 nucleotide-long single-stranded RNA molecules that act to inhibit gene expression by binding to target messenger RNAs (mRNAs). Because miRNAs bind to these targets with limited base pairing, it has been difficult to find targets. The goal of our study was to identify cellular mRNAs targeted by KSHV-encoded miRNAs. Microarray analysis of cells expressing the KSHV miRNAs revealed a set of 81 genes that were changed. Several genes are regulators of important functions such as blood vessel growth, cell proliferation, and cell death. One target, thrombospondin 1, is a potent inhibitor of blood vessel growth and is known to be down-regulated in Kaposi sarcoma tumors. Thrombospondin 1, which is targeted by multiple miRNAs, also showed reduced protein levels in our study. To our knowledge, our data describe the first targets for tumorvirus-encoded miRNAs and suggest that these novel regulators may have roles in pathogenesis.

## Introduction

Kaposi sarcoma–associated herpesvirus (KSHV) is the causative agent of Kaposi sarcoma (KS) and is associated with primary effusion lymphoma (PEL) and a subset of multicentric Castleman disease [1–4]. In KS tumors and PELs, the majority of cells are latently infected and express only a subset of viral genes located within the latency-associated region [5,6]. Recently, 12 microRNA (miRNA) genes have been identified within this region [7–9].

miRNAs are 19 to 23 nucleotide (nt)–long RNAs that post-transcriptionally regulate gene expression through selective silencing of target messenger RNAs (mRNAs). Precursor miRNAs are expressed as hairpin structures from transcribed RNA that are cleaved by Drosha, exported from the nucleus through Exportin 5, and subsequently processed by Dicer. Mature miRNAs are then incorporated into the RNA-induced silencing complex (RISC), which guides their binding to 3′UTRs of target mRNAs and sequesters them to processing bodies, ultimately leading to inhibition of translation and mRNA degradation (for review see [10]). Although target recognition for miRNAs is not completely understood, a seed sequence within the miRNA (nts 2 through 8) is known to be critical for binding and target recognition. In this manner, a single miRNA may regulate a large number of genes [11]. Human miRNAs have so far been found to regulate fundamental biological processes such as developmental pattern formation, hematopoiesis, apoptosis, and cell cycle control (for review see [12]).

**Figure 1:**
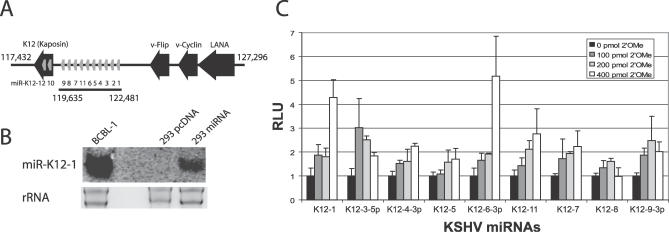
293 miRNA Cluster Cells Express KSHV miRNAs (A) Schematic diagram of the latency-associated region in the KSHV genome. The black bar indicates the miRNA cluster containing ten KSHV miRNA genes (grey bars) that were inserted into the expression vector. (B) Northern blot analysis of KSHV miRNAs in 293 pmiRNA cluster cells versus 293 pcDNA control cells and BCBL-1 cells. Total RNA (30 μg) was loaded and hybridized to a probe for miR-K12–1. Ribosomal RNA (rRNA) is shown as a loading control. (C) Luciferase de-repression assays show expression of all miRNAs within the cloned cluster in 293 pmiRNA cluster cells. The level of 2′OMe RNA was kept constant at 400 pmol with filler 2′OMe targeting miR-K12–10, a KSHV miRNA not represented in the cluster. Luciferase sensor (200 ng) was transfected along with the noted amounts of 2′OMe RNA specific to that miRNA. Transient transfection assays were done twice in triplicate and luciferase activity was normalized to total protein concentration. RLU, relative light units.

miRNAs have been identified within several DNA viruses, including herpesviruses (for reviews see [13–15]). A total of 17 miRNAs, encoded by 12 miRNA genes, have been cloned from KSHV-infected PEL cells, and interestingly, all are located within the KSHV latency-associated region (Figure 1A). This region encodes the latency-associated nuclear antigen (LANA), v-Cyclin, v-Flip, and the kaposin gene family, all of which modulate host cellular gene expression and signal transduction in latently infected cells [6,16–21]. We hypothesize that KSHV-encoded miRNAs target host/cellular gene expression and, as a result, play a role in viral pathogenesis.

As viral miRNAs have no sequence conservation to metazoan miRNAs, we are able to study them through ectopic expression without interfering with endogenous miRNAs. In this study, we have stably expressed ten KSHV miRNA genes in 293 cells and identified a total of 81 gene expression changes by microarray expression profiling. We confirmed a subset of these by quantitative real-time (qRT)–PCR, luciferase knockdown experiments, and Western blot analysis. Importantly, we identified thrombospondin 1 (THBS1), a potent inhibitor of angiogenesis reported to be down-regulated in KS lesions [22], as a target of multiple KSHV miRNAs.

## Results

### Ectopic Expression of KSHV miRNAs in 293 Cells

KSHV miRNAs are coordinately expressed with the latency-associated genes *LANA, v-Cyclin, v-Flip,* and *kaposin,* which all modulate the host cellular environment in KS tumors cells [6,16–21]. Hence, we hypothesized that KSHV-encoded miRNAs target host/cellular gene expression and as a result play a role in viral pathogenesis.

To identify potential miRNA targets without interference from viral proteins, we introduced the miRNA cluster, which encodes ten miRNA genes within a 2.8-Kbp (kilo–base pair) region (nts 119,635 to 122,481; Figure 1A), into a cytomegalovirus promoter containing expression plasmid and stably expressed the resulting construct or empty vector control in 293 cells. Cell populations rather than single-cell clones were used for subsequent experiments to avoid the risk of detecting expression differences caused by specific integration events.

To confirm miRNA expression in stable 293 pmiRNA cluster cells, we performed Northern blot analysis and luciferase reporter de-repression assays. The expression level of miR-K12–1 was about 20% compared to latently infected PEL cells, which contain a high copy number of KSHV episomes (Figure 1B). To detect individual miRNA expression, we performed luciferase reporter de-repression assays. First, sensor constructs each containing two complementary binding sites for a specific miRNA within the 3′UTR of pGL3 were constructed for each miRNA. Co-transfection of these constructs with individual miRNA expression vectors showed a dose-dependent inhibition, confirming sensor specificity (Figure S1). Individual KSHV miRNA expression in stable 293 pmiRNA cluster cells was examined using 2′OMe RNA antagomirs against each miRNA, which inhibit miRNA-containing RISC complexes in a sequence-specific manner [23,24]. As seen in Figure 1C, all nine miRNA sensor constructs show a dose-dependant de-repression between 2- and 5-fold in the presence of 2′OMe RNAs, confirming miRNA expression from the cluster.

### Expression Profiling Revealed 81 Gene Expression Changes in miRNA-Expressing Cells

For each cell line, 293 pmiRNA cluster and 293 vector control, two independent cultures were harvested at two time points for a total of eight samples. RNA extraction, quantification, cRNA synthesis, hybridization, and washing steps were done as recommended by the manufacturer and as previously described [16,25].

**Figure 2:**
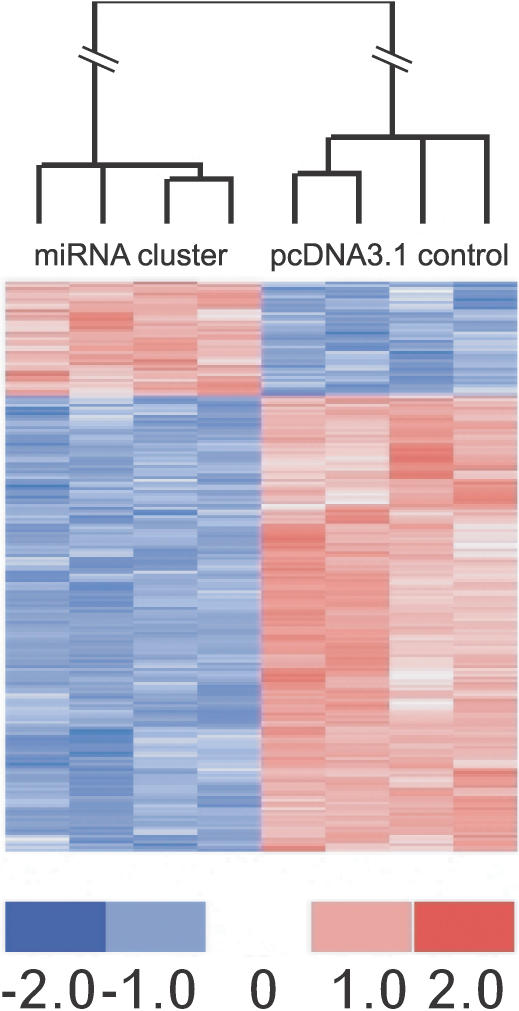
miRNA Responsive Gene Expression Profiles Colors represent changes in variance-normalized gene expression differences for individual genes represented by the probe sets as indicated on the color scale. The dendrogram denotes the relative relationship among the significant probe sets (*p* < 0.001) among the eight samples.

Figure 2 shows the expression profile of genes found to be significantly changed (*p* < 0.001) between both cell lines. Probe sets (205) were identified representing 177 genes. Of the 177 expression differences, the majority (137) showed decreased expression, while 39 genes showed expression increases. A cross validation (CV) analysis showed that of the 205 initial probe sets, a total of 81, representing 73 genes with 3′UTRs, show 100% CV (Table S1). This analysis, based on *t*-values of signal intensities, gives statistically robust data but includes probe sets with relatively small fold changes. For the final data set, 65 genes were decreased in their expression between 1.05- and 20.4-fold, while eight genes were increased between 1.05- and 1.4-fold (Table S1). Eight genes showed decreases greater than 4-fold. Interestingly, five of these genes—*SPP1* (osteopontin), *THBS1, S100A2* (S100 calcium binding protein A2), *PRG1* (plasticity related gene 1) (now named *SRGN*), and *ITM2A* (integral membrane protein 2A)—have roles in processes such as proliferation, immune modulation, angiogenesis, and apoptosis.

**Table 1:**
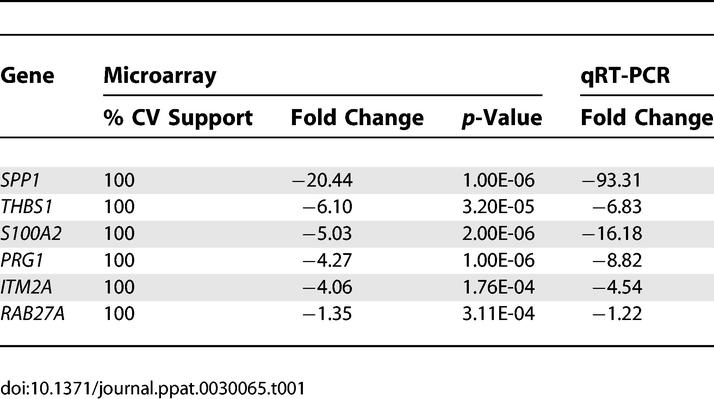
Fold Changes for Selected Genes

### qRT-PCR and Bio-Statistical Analyses Validate Potential Targets

Based on the CV support and high fold changes, we chose six genes for qRT-PCR analysis. As seen in Table 1, the fold changes observed with qRT-PCR closely match and validate the microarray results.

To determine whether the microarray gene changes are due to direct miRNA targeting, we scanned the 3′UTRs of the affected genes for potential miRNA binding sites. We initially concentrated on the seed sequences of the miRNAs (nts 2 through 8), which are known to be critical determinants of miRNA target recognition [11]. A similar analysis showed that the presence of seed matches within 3′UTRs for tissue-specific miRNAs predicted low expression levels for the corresponding genes [26]. In our analysis, we first determined the number of perfect and single mismatch seed matches for each miRNA by scanning a total of 222,817 nts representing 116 3′UTRs compiled from the Ensembl database (http://www.ensembl.org). As controls, all 3′UTR sequences were randomly shuffled ten times, creating unrelated sequences with conserved guanine-cytosine content, and seed match scans were repeated for each shuffle. As an additional control, the 116 3′UTRs were screened for Epstein Barr virus (EBV)–encoded miRNA seed binding sites. EBV miRNAs lack sequence homology to both human and KSHV miRNAs. The frequency for perfect KSHV miRNA seed matches was one in every 865 nts, compared to 1,358 nts for shuffled sequences and 1,400 nts for unrelated EBV miRNAs (Table 2). The observation that perfect seed matches for KSHV miRNAs are significantly (*p* < 0.05) enriched within the 3′UTRs of genes identified by microarray analysis indicates that these genes are targeted by miRNAs.

**Table 2:**
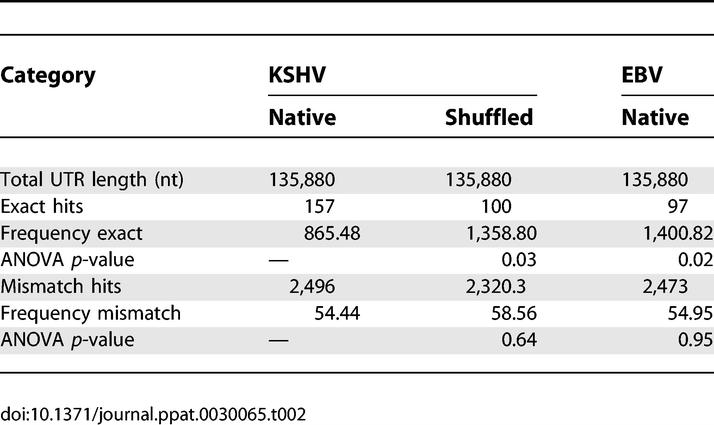
Seed Matching Data Using an Ad Hoc Scanning Algorithm

3′UTRs of *SPP1, PRG1, ITM2A, S100A2, RAB27A,* and *THBS1* were also scanned by miRanda [27], which predicted high probability miRNA binding sites (scores above 190 and/or free energy values below −20 kcal/mol) within their 3′UTRs (Figure S3). In particular, *THBS1* was predicted to have 34 binding sites for 12 KSHV miRNAs. Together, the bio-statistical analyses and the confirmation by qRT-PCR strongly suggest that the observed gene expression changes are due to viral miRNA expression.

**Figure 3:**
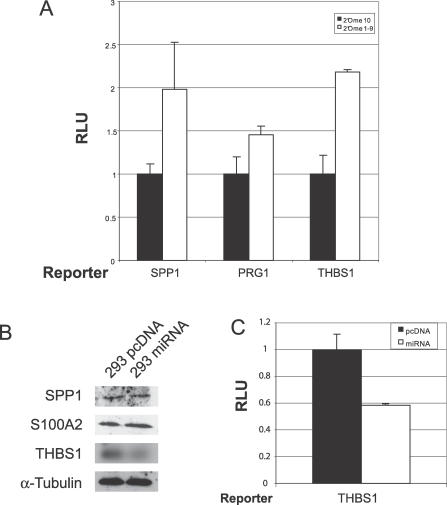
KSHV miRNAs Down-Regulate Targets (A) Luciferase de-repression assays were performed in 293 pmiRNA cluster cells using 200 ng of sensor vectors containing the 3′UTRs of *SPP1, PRG1,* or *THBS1*. Then, 400 pmol of either the 2′OMe miR-K12–10 (control) or a mixture of 2′OMe RNA (specific to miR-K12–1–miR-K12–9 and miR-K12–11) (40 pmol each) were added to each transfection. (B) Western blot analysis of 293 pmiRNA cluster cells versus 293 pcDNA control cells. (C) Transient luciferase assay in BJAB cells using 4.8 μg of either pcDNA3.1 empty vector or miRNA cluster expression vector and 200 ng of *THBS1* luciferase sensor vector.

To test this directly, we inserted the 3′UTRs of *SPP1*, *PRG1*, or *THBS1* into the pGL3 promoter vector downstream of the luciferase gene. Co-transfection of these constructs into 293 pmiRNA cluster cells with a mixture of ten 2′OMe RNAs resulted in significant de-repression of luciferase activity as compared with an unrelated control 2′OMe RNA (Figure 3A). These results show that the 3′UTRs of *SPP1*, *THBS1*, and *PRG1* confer miRNA-dependent repression to a heterologous reporter, further supporting that the observed expression differences are miRNA-dependent.

### Protein Levels of THBS1 Are Greatly Reduced in the Presence of KSHV miRNAs

Western blot analysis was performed to determine whether decreased mRNA levels for *SPP1, S100A2,* and *THBS1* translate into inhibition of translation and, subsequently, decreased protein abundance. As seen in Figure 3B, THBS1 expression was dramatically reduced (>10-fold) in KSHV miRNA-expressing 293 cells when compared to vector control. SPP1 and S100A2 protein levels were not affected even though mRNA levels were decreased 20.4- and 5.1-fold, respectively. Hence, for one out of three genes examined by Western blot, we detected reduced protein levels in miRNA- expressing cells.

### KSHV miRNAs Repress *THBS1* in BJAB Cells

To ensure that KSHV miRNA-mediated repression of *THBS1* is not a 293 cell line specific effect, we performed transient transfection assays in BJAB cells. Either vector control or miRNA cluster was transfected into BJAB cells along with the *THBS1* luciferase reporter. Luciferase readouts showed a 2-fold reduction of luciferase in response to expression of the miRNAs (Figure 3C). Thus, targeting of *THBS1* by KSHV-encoded miRNAs can be observed in 293 cells and in BJAB, a lymphoid cell line.

### *THBS1* Is Targeted by Multiple KSHV miRNAs

**Figure 4:**
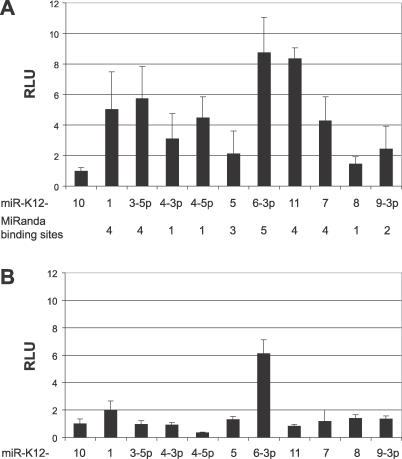
*THBS1* Is Targeted by Multiple KSHV miRNAs (A) Luciferase de-repression assays were performed in 293 pmiRNA cluster cells with 200 ng of *THBS1* 3′UTR luciferase sensor vector and 400 pmol of individual 2′OMe RNAs as indicated. Lysates were assayed at 72 h. The number of miRanda-predicted high affinity binding sites for each miRNA are listed below. (B) The same luciferase de-repression assays were performed as in (A) except with 200 ng of *HS6ST2* 3′UTR luciferase sensor vector.

We next asked whether specific KSHV miRNAs may be responsible for decreased THBS1 expression levels. We performed luciferase de-repression assays by co-transfection of the *THBS1* 3′UTR reporter and 2′OMe RNAs specific to individual KSHV miRNAs. Our results demonstrate that *THBS1* is targeted by multiple KSHV miRNAs; in particular, miR-K12–1, miR-K12-3-3p, miR-K12-6-3p, and miR-K12–11 lead to strong de-repression of the reporter (Figure 4A). Interestingly, the observed de-repression levels closely match with the number of miRanda-predicted high affinity binding sites (Figure S3) in that the four miRNAs with the highest levels of repression also have the highest number of predicted binding sites. It is important to note that we observed higher levels of de-repression than those seen in Figure 3A. However, although the total amount of 2′OMe is the same in both experiments, the concentrations of each individual 2′OMe is 10-fold greater for Figure 4. As an additional control we tested the 3′UTR of *HS6ST2* in the same assay (Figure 4B). The 3′UTR of *HS6ST2* is 2,213 bp in length (compared to 2,095 for *THBS1*), is shown to have no expression change in response to the KSHV miRNAs via microarray, and was predicted by miRanda to have few miRNA binding sites (unpublished data). As shown in Figure 4B, *HS6ST2* showed virtually no difference in luciferase activity for all KSHV miRNAs, with the exception of miR-K12-6-3p. These results show that the inhibition of *THBS1* by the KSHV miRNAs is specific. The reason for the 6-fold de-repression with miR-K12-6-3p is not clear as there is only one high affinity binding site predicted for *HS6ST2;* however, the de-repression shown with *THBS1* is still greater than this. Thus, our data suggest that *THBS1* is targeted by multiple KSHV miRNAs rather than a specific, individual viral miRNA.

### Decreased THBS1 Protein Levels Translate into Reduced TGF-β Activity

**Figure 5:**
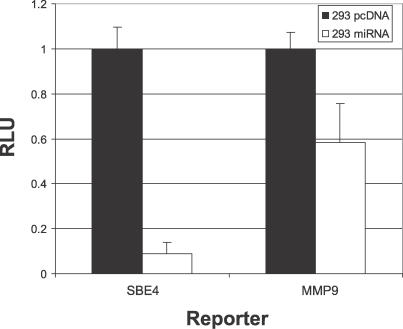
Decreased THBS1 Protein Levels Translate into Reduced TGF-β Activity Luciferase reporters containing Smad binding sites *(SBE4)* or the *MMP-9* promoter (200 ng) were transfected into 293 pmiRNA cluster or 293 pcDNA control cells and analyzed for luciferase activity at 72 h.

THBS1 is a matricellular glycoprotein with strong anti-proliferative and anti-angiogenic activity that can activate the latent form of TGF-β by direct binding through its RFK and WxxW motifs [28,29]. We asked whether TGF-β activity is decreased in cells that express KSHV miRNAs and, as a result, have decreased levels of THBS1. To this end, we performed transient transfection assays using two different TGF-β–responsive promoters (Figure 5). SBE4 contains four Smad-binding elements and is commonly used to detect active TGF-β [30]. We also tested the native matrix metalloproteinase-9 promoter *(MMP-9),* which is activated in response to TGF-β signaling [31]. Reporter vectors were transfected into 293 pmiRNA cluster or vector control cells and cell lysates assayed for luciferase activity. Transcription from the SBE4 reporter was reduced 11-fold in cells expressing KSHV miRNAs, while *MMP-9* promoter activity was inhibited 2-fold. It is important to note that transcript levels of both *TGF-β* and *Smad3* were not altered as determined by gene expression profiling; thus, the decreased TGF-β activity is not due to miRNA-dependent inhibition of TGF-β or Smad3. These data demonstrate that decreased THBS1 protein levels translate into downstream TGF-β signaling.

## Discussion

miRNA genes have been identified in all herpesviruses studied to date, and an important question remains as to how these novel regulators contribute to viral biology. Viral miRNAs may target viral and/or host cellular gene expression. The fact that miRNAs are not conserved between virus families suggests that each virus may have evolved to target diverse sets of genes to aid its specific replication strategy (i.e., tissue tropism for latent, lytic, and persistent infection). Observations made on genetically modified KSHV viruses suggest that most KSHV miRNAs are not required for lytic replication in culture [32]. We hypothesized that KSHV miRNAs, which are coordinately expressed within the latency-associated region, modulate host cellular gene expression to promote an environment conducive to latency and persistence in the infected host.

One of the most problematic areas of miRNA biology is identifying their targets and functions. Due to the rather large number of human miRNAs and the limited homology requirements for binding, *in silico*–based attempts for miRNA target predictions have suggested a model by which thousands of genes are targeted [11]. However, in contrast to the genetically defined roles of some *Caenorhabditis elegans* and Drosophila melanogaster miRNAs, only a few vertebrate miRNA targets have been determined [10,12,33].

This study aimed to experimentally determine cellular genes targeted by virally encoded miRNAs. Ectopic expression of ten KSHV miRNA genes in 293 cells resulted in significant (*p* < 0.001) expression differences of 81 genes, 65 of which were down-regulated (Table S1). These results were confirmed by qRT-PCR and bioinformatics analyses, which revealed a significant enrichment for seed sequence matches within the 3′UTRs of genes changed (Tables 1 and 2). Interestingly, the number of seed matches in our analyses varied significantly between miRNAs. For example, miR-K12–1, K12-6-5p, and K12-9-5p show significantly increased hits and therefore may target more genes (Figure S2). In a related study, Sood et al. recently demonstrated that this seed sequence analysis is effective at predicting decreased expression levels for genes containing seed binding sites to tissue-specific miRNAs [26].

While the majority of observed gene changes were moderate decreases, eight genes showed greater than 4-fold down-regulation, including *SPP1, S100A2, ITM2A, PRG1,* and *THBS1* (Table 1). We (i) confirmed KSHV miRNA-dependent inhibition of the 3′UTRs of *SPP1, PRG1,* and *THBS1* using luciferase de-repression assays in the 293 miRNA cluster cells, (ii) demonstrated inhibition of *THBS1* in BJAB cells, and (iii) found decreased protein levels for THBS1 in the presence of KSHV miRNAs (Figure 3). Additionally, we observed that *THBS1* is targeted by multiple KSHV miRNAs, with the major miRNA species being miR-K12–1, miR-K12-3-3p, miR-K12-6-3p, and miR-K12–11 (Figure 4A). It is important to note that the observed effects were specific to *THBS1* since the 2,213-bp *HS6ST2* 3′UTR, although of comparable length, was not similarly de-repressed. Multiple miRNAs have previously been reported to target a single 3′UTR. Stark et al. found >50% of all predicted miRNA targets contain binding sites for more than one miRNA and that as many as 12 different miRNAs can target a single 3′UTR [34]. Generally, genes that require strict regulation have longer 3′UTRs and thus more binding sites for miRNAs. Additionally, for *THBS1,* miRanda [27] predicted 34 high probability binding sites for 12 different KSHV miRNAs within the *THBS1* 3′UTR (see Figure S3). These bioinformatic predictions are well in agreement with the levels of de-repression seen for individual miRNAs. The fact that *THBS1* has a long (2,095 bp) 3′UTR and has binding sites for multiple KSHV miRNAs suggests that inhibition of THBS1 is important for KSHV biology.

To investigate the biological effects of altered THBS1 levels, we utilized TGF-β–responsive reporter constructs and demonstrated decreased TGF-β activity (Figure 5). It is important to note that transcript levels of *TGF-β* and *Smad3* were not altered in response to miRNA expression; thus, the decreased TGF-β activity is not due to miRNA inhibition of TGF-β or Smad3.

Of those genes exhibiting the highest down-regulation in response to miRNAs, five are involved in proliferation, immune modulation, angiogenesis, and apoptosis pathways often altered in cancer. SPP1 is a secreted phosphoprotein also called osteopontin, which is known to interact with a variety of integrins as well as with CD44, and plays a role in cell-mediated immunity. SPP1 also has demonstrated anti-proliferative activity against virally infected mucosal epithelial cells [35,36]. S100A2 is a calcium-binding protein that is down-regulated in many tumor types and is thought to possess tumor suppressor activity. Additionally, S100A2 interacts with the p53 family proteins p67 and p73 and increases transcriptional activity of p53 [37,38]. ITM2A is an integral membrane protein that may play a role in selection of T cells in the thymus and may also function in cartilage development [39]. PRG1 is a granule proteoglycan secreted by hematopoietic cells and is involved in apoptosis [40].

THBS1 is a matricellular protein that functions in cell–cell and cell–matrix adhesion and is down-regulated in a number of human cancers. THBS1 possesses both anti-proliferative and anti-angiogenic activity [28,41]. Angiogenesis is a hallmark of KS tumors, and the inhibition of THBS1 would aid not only this process but also proliferation of KSHV-infected endothelial-derived KS tumor cells. Interestingly, Tarabolleti et al. reported that THBS1 expression is suppressed in KS lesions [22]. However, to date, this observation has not been linked to viral protein expression in KS lesions. THBS1 is also a strong immune stimulator that can recruit monocytes to sites of vascular injury [42] and regulate T cell migration through extracellular matrix [43]. Thus, down-regulation of THBS1 may also aid in immune evasion of KSHV-infected cells. While such connections are tempting, we realize the limitations of examining the effects of viral miRNAs on a single cell line. However, 293 cells support both latent and lytic KSHV infection in vitro, and their high transfection efficiency permitted experimental confirmation of a limited number of targets by using miRNA-specific reporter assays. We have also shown that THBS1 is inhibited in a lymphoid cell line, BJAB, in a transient assay, indicating that our results are not restricted to the 293 cell line.

In conclusion, this study yielded a starting list of potential KSHV miRNA targets, which warrants a further detailed analysis in all cell types infected with KSHV in vivo (lymphoid, epithelial, and endothelial cells). Our approach to identify miRNA targets by ectopic expression, gene expression profiling, reporter assays, and bio-statisticial analysis has revealed cellular targets with potential roles in KS biology. These data strongly suggest that virally encoded miRNAs may directly contribute to pathogenesis and potentially tumorigenesis in the infected host.

## Materials and Methods

### Plasmid construction, expression vectors, and miRNA sensor vectors.

For generation of pmiRNA cluster, a region from 119,635 to 122,481 nt within the KSHV genome [44] was amplified from BCBL-1 genomic DNA and inserted into pcDNA3.1/V5/HisA (Invitrogen, http://www.invitrogen.com). For plasmids expressing individual miRNAs, a region of approximately 200 nts surrounding each pre-miRNA hairpin was amplified from the cluster-containing plasmid and inserted into pcDNA3.1/V5/HisA. Stable 293 cell lines were generated by Effectene (Qiagen, http://www.qiagen.com) transfections followed by G418 selection for 4 wk at 100 μg/ml.

Luciferase reporter plasmids were created using the pGL3 promoter vector from Promega (http://www.promega.com). Synthetic oligonucleotides containing two complete complimentary copies of a miRNA sequence separated by a 9-bp-long spacer were inserted into the 3′UTR of the luciferase gene upstream of the poly-adenylation signal. All primer sequences are annotated in Table S2.

### Reporter assays using miRNA sensor vectors and 2′OMe RNA.

Lipofectamine 2000 (Invitrogen) was used to transfect luciferase reporter constructs with or without miRNA expressing vectors or 2′OMe RNA (Dharmacon, http://www.dharmacon.com) complementary to a miRNA of interest. Next, 4 × 10^5^ cells were seeded into a 6-well plate, transfected, and then incubated for 72 h. Cells were lysed with 250 μL of Cell Lysis Buffer (Promega) and 10 μL of lysate assayed for luciferase activity (Promega). Light units are normalized to total protein, determined using the BCA protein assay kit (Pierce, http://www.piercenet.com) according to manufacturer's instructions. All transient transfection experiments were performed twice in triplicate.

### Affymetrix array-based gene expression profiling.

Microarray experiments were performed using 293 cells stably transfected with pmiRNA cluster or vector control. For each cell line, four independent cultures of 10-cm plates at 80% confluence were used for RNA isolation.

RNA isolation was performed using the RNeasy kit as directed by the manufacturer (Qiagen). RNA was labeled using the GeneChip Eukaryotic One-Cycle Target Labeling Assay as directed by Affymetrix (http://www.affymetrix.com). Labeled target cRNA was used to interrogate Affymetix U133 2.0 plus human GeneChips that were hybridized for 16 h at 45 °C. After hybridization, the chips were washed and stained using Affymetrix fluidics protocol EukGE-WS2v5_450.

Arrays were scanned with an Affymetrix GeneChip 3000 scanner and normalization of signal intensity was performed using dChip [45]. The expression level was modeled using the perfect match only model.

Probe sets whose hybridization signal intensities exhibited a significant difference (*p* < 0.001 using a random variance model) between pmiRNA cluster and pcDNA control cells were identified using algorithms implemented in BRB ArrayTools developed by Dr. Richard Simon and Amy Peng Lam (http://linus.nci.nih.gov/BRB-ArrayTools.html). A hierarchical clustering algorithm was used to visually display the expression profiles of the probe sets found to be significant between the groups.

### qRT-PCR, Northern, and Western blot analysis.

RNA was reverse-transcribed using SuperScript III Reverse Transcriptase (Invitrogen) in the presence of random hexamers according to the manufacturer's protocols. qRT-PCR was performed using an Opticon II (MJ Research, http://www.bio-rad.com) and Dynamo HS SYBR-Green qPCR kit using cycling conditions as recommended by kit manufacturer (Finnzymes, http://www.finnzymes.fi). Primers were designed across exon boundaries using Vector NTI (Invitrogen) and are provided in Table S2. PCR signals were normalized to β-actin and fold changes reported as 2^ΔΔCt^. For Northern blot analysis, 30 μg of total RNA was loaded onto 12% 8M urea acrylamide gel and transferred onto Genescreen Plus. Probe labeling was performed using the StarFire oligonucleotide labeling system (IDT, http://www.idtdna.com).

Immunoblotting was performed as previously described [16]. All primary antibodies were purchased from Santa Cruz Biotechnology (http://www.scbt.com). HRP-conjugated secondary antibodies were purchased from Jackson ImmunoResearch (http://www.jacksonimmuno.com) and blots were developed with PicoWest substrate (Pierce).

### Bio-statistical data analysis.

The 3'UTR sequences of 116 genes were obtained from Ensembl (http://www.ensembl.org). 3′UTRs were then analyzed to extract all potential miRNA binding sites using an ad-hoc scanning program specifically developed to look at seed match binding. The program looks for occurrences of any number of user-provided patterns, each of which represents a target for a different miRNA, and accepts both exact matches or near-exact matches (with at most one mismatched nucleotide). For each sequence, the program returns a list containing the positions of the exact matches, and a second list containing the positions of the near-exact matches and the position of the mismatched base within each matched subsequence.

## Supporting Information

Figure S1 All KSHV miRNA Luciferase Sensor Vectors Are Responsive to Their Respective miRNAs Luciferase sensor (100 ng) was co-transfected with the noted amounts of the single miRNA expression vectors. The total amount of DNA transfected was kept constant with filler pCRII vector (Invitrogen).
(536 KB AI)

Figure S2 Distribution of Seed Matches for KSHV miRNAs within the 3′UTRs of Analyzed Genes Using an ad hoc scanning algorithm, the 3′UTR of 205 genes were scanned for seed sequence binding sites of all miRNAs within the cluster for (A) exact seven nucleotide matches or (B) allowing one mismatch.
(548 KB AI)

Figure S3 mirRanda-Predicted Binding Sites for the KSHV miRNAs within the 3′UTRs of *THBS1, ITM2A, SPP1, PRG1,* and *S100A2*
3′UTR sequences were obtained from Ensembl. Scans were performed with the following parameters: Gap Open Penalty, −2; Gap Extend, −8; Score Threshold, 160; Energy Threshold, −12; Scaling Parameter, 4. Hits were included that either had energy threshold less than −18 kcal/mol or scores above 190.
(73 KB DOC)

Table S1 List of Changed Genes with 100% CV (228 KB DOC)

Table S2 List of Primers for the Cloning of miRNA Expression and Sensor Constructs (56 KB DOC)

### Accession Numbers

Ensembl (http://www.ensembl.org) accession numbers for genes listed in this paper are *HS6ST2* (ENST00000370837), *ITM2A* (ENST00000373298), *PRG1* (ENST00000242465), *RAB27A* (ENST00000336787), *S100A2* (ENST00000368711), *SPP1* (ENST00000237623), and *THBS1* (ENST00000260356). The Entrez Nucelotide (http://www.ncbi.nlm.nih.gov/entrez/query.fcgi?db=Nucleotide) accession number for the KSHV miRNA cluster is AY973824. The Gene Expression Omnibus (http://www.ncbi.nlm.nih.gov/projects/geo) accession number for the microarray data is GSE7554.

## Abbreviations

bp: base pair
CV: cross validation
EBV: Epstein Barr virus
KSHV: Kaposi sarcoma–associated herpesvirus
miRNA: microRNA
mRNA: messenger RNA
nt: nucleotide
PEL: primary effusion lymphoma
qRT-PCR: quantitative real-time PCR
THBS1: thrombospondin 1
