# Supplementary figures and images for: Identification of Cellular Genes Targeted by KSHV-Encoded MicroRNAs
Source: PLoS Pathog. 2007 May 11;3(5):e65. doi: 10.1371/journal.ppat.0030065 (PMC1876501; doi:10.1371/journal.ppat.0030065)

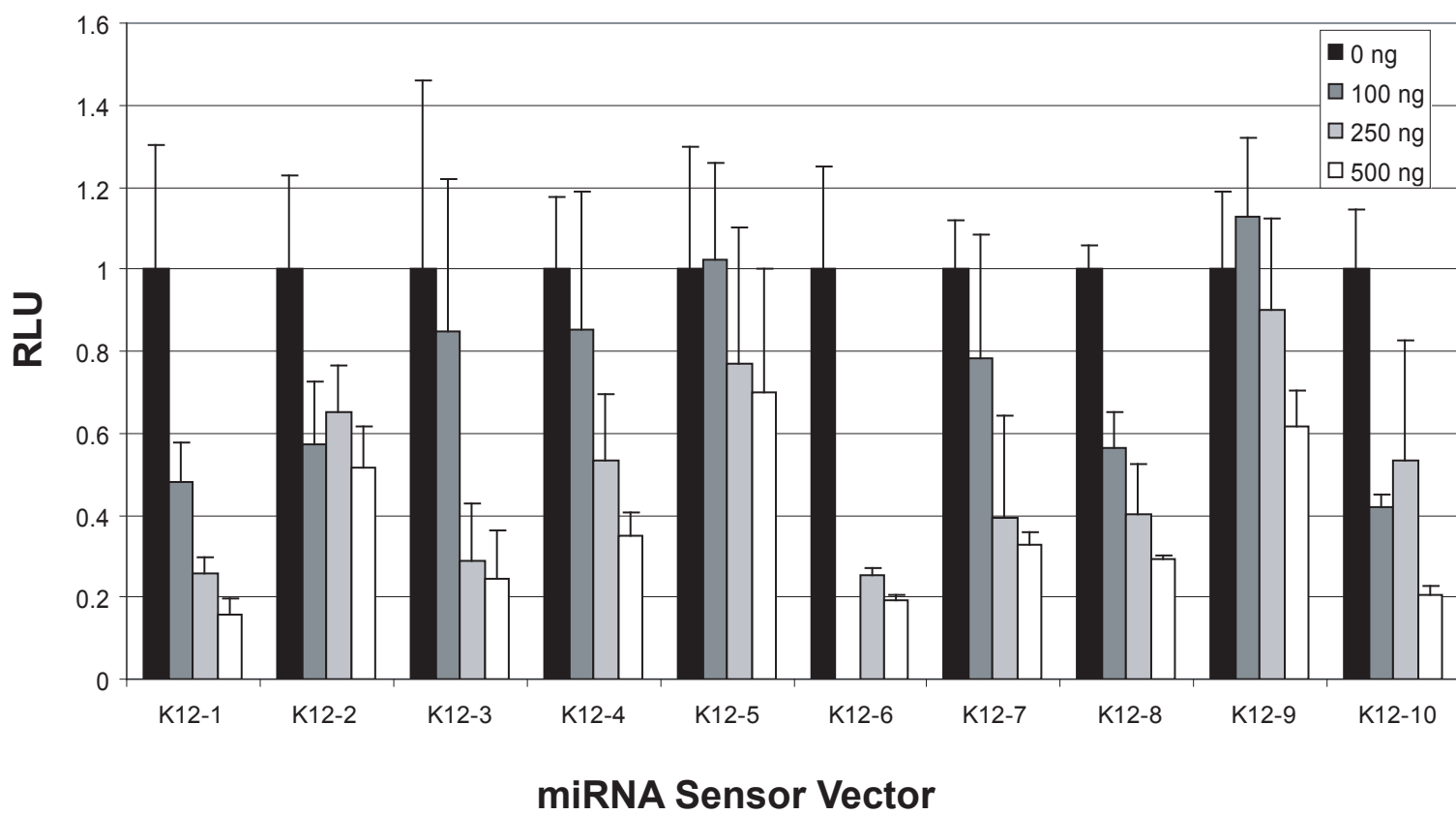

Supplement: Figure S1 — Luciferase sensor (100 ng) was co-transfected with the noted amounts of the single miRNA expression vectors. The total amount of DNA transfected was kept constant with filler pCRII vector (Invitrogen). (536 KB AI) [file ppat.0030065.sg001.pdf]

**A**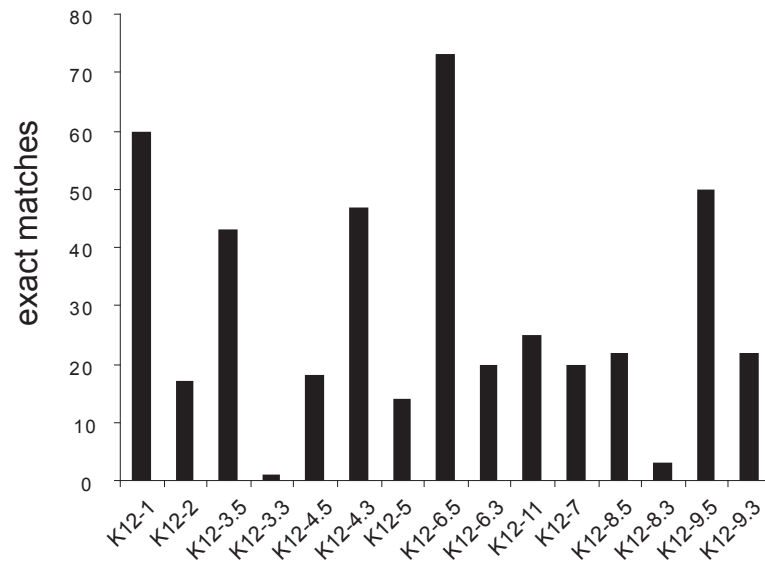**B**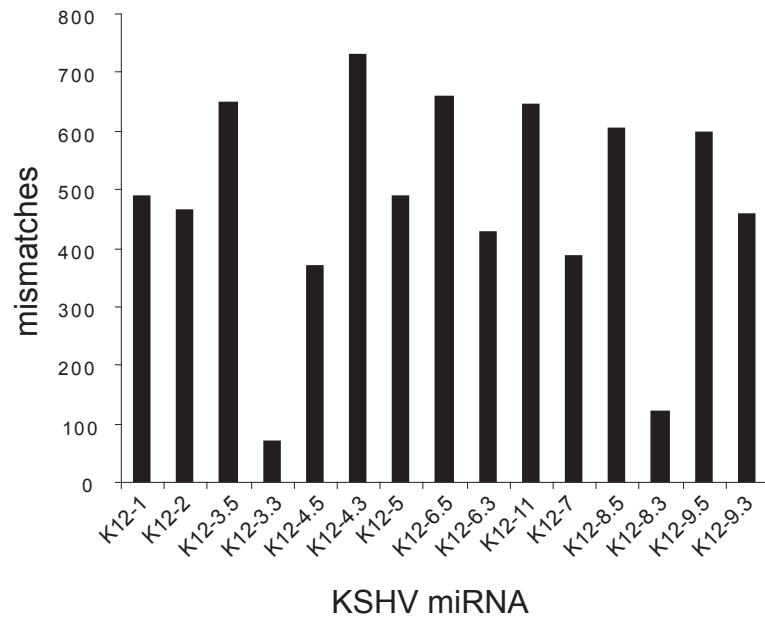

Supplement: Figure S2 — Using an ad hoc scanning algorithm, the 3′UTR of 205 genes were scanned for seed sequence binding sites of all miRNAs within the cluster for (A) exact seven nucleotide matches or (B) allowing one mismatch. (548 KB AI) [file ppat.0030065.sg002.pdf]
