# Supplementary material for: Identification of Cellular Genes Targeted by KSHV-Encoded MicroRNAs
Source: PLoS Pathog. 2007 May 11;3(5):e65. doi: 10.1371/journal.ppat.0030065 (PMC1876501; doi:10.1371/journal.ppat.0030065)
Supplement: Figure S3 — 3′UTR sequences were obtained from Ensembl. Scans were performed with the following parameters: Gap Open Penalty, −2; Gap Extend, −8; Score Threshold, 160; Energy Threshold, −12; Scaling Parameter, 4. Hits were included that either had energy threshold less than −18 kcal/mol or scores above 190. (73 KB DOC) [file ppat.0030065.sg003.doc]

**THBS1**

**K12-11**

Score: 190

Energy: -21.24 kCal/Mol

miRNA: 3' AG-C-CU-GUGUCC-GAUUCGUAAUU 5'

| | || || ||| | ||||| |||

THBS1: 5' ACGGGGAGCAGAGGCCAAAGCACTAA 3'

Score: 208

Energy: -14.72 kCal/Mol

miRNA: 3' AGCCUGUGUC-C-GAUUCGUA-AUU 5'

|||| | || | |||||||| |||

THBS1: 5' GCGGA-A-AGAGTTTAAGTGTCTAA 3'

Score: 193

Energy: -16.75 kCal/Mol

miRNA: 3' AG-CC-UGU-GUCCGA-UUCGUAAUU 5'

|| || | | | |||| ||| ||||

THBS1: 5' TCTGGAAGATCTGGCTGAAGGATTAT 3'

Score: 191

Energy: -12.39 kCal/Mol

miRNA: 3' AGCCUGUGUCCGAUUCGUAA-UU 5'

|| | |||| || |||||| ||

THBS1: 5' TCAAAAACAGACTCAGCATTCAG 3'

**K12-9**

Score: 168

Energy: -18.27 kCal/Mol

miRNA: 3' AA-U-G-CG-U-CGACGCAUAUGGGUC 5'

| | | || || | |||||||||

THBS1: 5' ATAAGCTGCTCTGCCCCTTGTGCTCAG 3'

Score: 197

Energy: -13.31 kCal/Mol

miRNA: 3' AAUGCGUCGACG-CAU-AUGGGUC 5'

||| |||| | ||| ||||||

THBS1: 5' TTA-A-AGCTACTGTAGTACCTAA 3'

**K12-8**

Score: 194

Energy: -23.29 kCal/Mol

miRNA: 3' G-CACGAGA-G-AGUCA-G-C-GCGGAU 5'

| ||||| | | ||||| | | | ||||

THBS1: 5' CTGTGCT-TGCATCAGTGTGGACTCCTA 3'

**K12-7**

Score: 202

Energy: -20.46 kCal/Mol

miRNA: 3' UCG-CGGUCGUUGUA-CCC-UAGU 5'

||: ||||| ||| | ||| ||||

THBS1: 5' AGTGGCCAG-AAT-TAGGGAATCA 3'

Score: 169

Energy: -18.79 kCal/Mol

miRNA: 3' UCGCGGUCGUUGU-ACCCUAGU 5'

| :||| |||| |||| ||

THBS1: 5' TCCTTCAGGAACACGGGGAGCA 3'

Score: 196

Energy: -15.44 kCal/Mol

miRNA: 3' UCGCGGUCGUU-GU-AC-CCU-A-G-U 5'

|:||: || | || || ||| | | |

THBS1: 5' TGTGCTTGC-ATCAGTGTGGACTCCTA 3'

Score: 194

Energy: -13.56 kCal/Mol

miRNA: 3' UCGCGGU-C-G-UU-GUAC-CCU-AGU 5'

|| |||| | :| || | ||| |||

THBS1: 5' AG-GCCATCTCTGAGCA-GTGGACTCA 3'

**K12-6-5p**

Score: 215

Energy: -22.65 kCal/Mol

miRNA: 3' GGCUACCU-AA-U-CCACGAC-GACC 5'

::| |||| || | | ||||| ||||

THBS1: 5' TTGCTGGATTTCATGATGCTGACTGG 3'

Score: 197

Energy: -19.13 kCal/Mol

miRNA: 3' GG-C-UA--CC-U-A--AUCC-AC-G-A-C-GACC 5'

|| | :| || | | | || || | | | ||||

THBS1: 5' CCAGTGTAAGGCAGTGCT-GGCTGCCATTGCCTGG 3'

**K12-6-3p**

Score: 223

Energy: -16.54 kCal/Mol

miRNA: 3' G-A-G-U-U-GUCG-G-G-CUUUUGGU-AG-U 5'

| | | | | |||| | |||||||| || |

THBS1: 5' CTTGCAAGAACAGCACAAGGAAAATCAGTCTA 3'

Score: 201

Energy: -12.28 kCal/Mol

miRNA: 3' GAGU-UGUCGGGC-UUU-UGGUA-GU 5'

| | || | | ||| ||||| ||

THBS1: 5' GGAAGA-AG-C-GTAAAGACTATCCA 3'

Score: 200

Energy: -15.96 kCal/Mol

miRNA: 3' GAGUUGUCGGGCUUU-UGGUAGU 5'

:|||: :| ::|||| |||||||

THBS1: 5' TTCAGGGGATTGAAAGACTATTG 3'

Score: 194

Energy: -16.37 kCal/Mol

miRNA: 3' GAG-UUGUCG-GGCUUUUGGUAGU 5'

:|: ||: || ||||||||| |

THBS1: 5' TTTAAATTGCAAAGAAAGCCATGA 3'

Score: 192

Energy: -14.28 kCal/Mol

miRNA: 3' GA-GUUGUCGGGCUUUUGGUAGU 5'

:| |||::|| | ||||||||||

THBS1: 5' TTACAATGGCAC-AAAATTATTA 3'

**K12-5**

Score: 201

Energy: -19.78 kCal/Mol

miRNA: 3' G-G-C-CGU-U--C-A-AG-G-UCC-GUAG-GA-U 5'

| | | ||| : | | |: | ||| |||| || |

THBS1: 5' CACAGAGCAGGGTGCTATTGTGAGGCCATCTCTGA 3'

Score: 194

Energy: -16.88 kCal/Mol

miRNA: 3' GGCCGUUCAAGGUCCGUA-GGAU 5'

|| | ||:||||||| || |

THBS1: 5' AAAGC-ATTTTCAGGCATGTC-A 3'

Score: 191

Energy: -15.16 kCal/Mol

miRNA: 3' GG-CCGUUCAAGGUCCGUAGG-AU 5'

|| || || || ||||| ||| ||

THBS1: 5' CCTGG-AAATT-TAGGC-TTCATA 3'

**K12-4-5p**

Score: 201

Energy: -15.90 kCal/Mol

miRNA: 3' GGA-U-CU-C-A-UGA-CGCCAAAUCGA 5'

::| | || | | ||| || | ||||||

THBS1: 5' TTTCATGATGCTGACTGGC-G-TTAGCT 3'

**K12-4-3p**

Score: 214

Energy: -12.56 kCal/Mol

miRNA: 3' A-GUC-GAU-CCGGAGUCAU-AAGAU 5'

| | | ||: | |||||| |||||

THBS1: 5' TACTGCCTGTAGAGTTAGTATTTCTA 3'

**K12-3-5p**

Score: 208

Energy: -26.95 kCal/Mol

miRNA: 3' GCGAC-G-GCA-G-GAGUCUUACACU 5'

| ||| | | | : ||||||||| ||

THBS1: 5' CTCTGCCCCTTGTGCTCAGAGTG-GA 3'

Score: 202

Energy: -18.20 kCal/Mol

miRNA: 3' GC-G-A-C-G-G-C-A-GGAGUCUUACAC-U 5'

:| | | | : | | :|||||| |||| |

THBS1: 5' TGCCATTGGAATAGATATCTCAGATTGTGTA 3'

Score: 167

Energy: -20.25 kCal/Mol

miRNA: 3' GCG-A-CGGCAG-GAGUC-UUACACU 5'

:|: ||| || || || |||| ||

THBS1: 5' TGTGAGGCCATCTCTGAGCAGTG-GA 3'

Score: 201

Energy: -17.76 kCal/Mol

miRNA: 3' GCGACGG-C-AGGAGU-CUUAC-ACU 5'

:|||: | | :||| | ||| |||

THBS1: 5' TATTGCTGGAT-TTCATG-ATGCTGA 3'

**K12-3-3p**

Score: 191

Energy: -15.74 kCal/Mol

miRNA: 3' A-C-AGUGUAAGAC-ACUGG-CGCU 5'

| | |: ||| || ||||| |||

THBS1: 5' TGGATTTCATGATGCTGACTGGCGT 3'

Score: 190

Energy: -16.37 kCal/Mol

miRNA: 3' ACAGU-G-UAAGACACUGGCGC-U 5'

||| : :|| |||||||| | |

THBS1: 5' CCTCATTTGTTGTGTGACTGAGTA 3'

Score: 206

Energy: -14.540000 kCal/Mol

miRNA: 3' G-UC-UAGC-U-G-GG-CC-UGAUG-UC-AA 5'

| :| ||:| | | :| || ||||| || ||

THBS1: 5' CTGGTATTGCACCTTCTGGAACTATGGGCTT 3'

**K12-1**

Score: 208

Energy: -13.910000 kCal/Mol

miRNA: 3' CG-AAUGUGGGUCAAAGGACAUUA 5'

| || | :|: ||||||| |||

THBS1: 5' CCTTTTC-TCTTTTTTCCTG-AAT 3'

Score: 198

Energy: -13.800000 kCal/Mol

miRNA: 3' CGAAU-G-UGGGUCAA-A-GGA-CAUUA 5'

:||| : ||:| ||| | ||| | |||

THBS1: 5' ATTTATTAACTCTGTTCTGCCTGGAAAT 3'

Score: 193

Energy: -13.580000 kCal/Mol

miRNA: 3' CGAAUGUGGGUCAAA-GG-ACAUU-A 5'

|||| |:|| ||| || ||||| |

THBS1: 5' TATTAC-CTCA-TTTGTTGTGTGACT 3'

Score: 190

Energy: -13.540000 kCal/Mol

miRNA: 3' CGAAUGUG-GGUCAAAGG-AC-A-U-UA 5'

|||| | | :| ||||| || | | ||

THBS1: 5' GCTT-C-CTTC-TTTTCTGTGCTTGCAT 3'

**ITM2A**

**K12-1**

Score: 194

Energy: -12.50 kCal/Mol

miRNA: 3' CGAAUGU-GG-GUCAAAGG-A-CAUUA 5'

|| ||| : ||| ||| | |||||

ITM2A: 5' GC-AACAGATAGAGTGTCCTTGGTAAT 3'

**K12-6-5p**

Score: 198

Energy: -17.91 kCal/Mol

miRNA: 3' GGC-U-A-C-CUAAUCCACGACGACC 5'

::| | | | ||||||||| |||

ITM2A: 5' TTGCAGTTCTG-TTAGGTGCT-CTGT 3'

**K12-4-3p**

Score: 190

Energy: -13.76 kCal/Mol

miRNA: 3' AGUC-GAUCCGGA-GU-CAUAAGAU 5'

|| :|||| :| || | ||||||

ITM2A: 5' GGAGTTTAGG-TTGTATGAATTCTA 3'

**SPP1**

**K12-1**

Score: 190

Energy: -15.12 kCal/Mol

miRNA: 3' CGAAUG-U-GGGUCAAAGGA-C-A-UUA 5'

|||| | |:|||||| || | | |||

SPP1: 5' GCTT-CTTTCTCAGTTTATTGGTTGAAT 3'

**K12-6-3p**

Score: 196

Energy: -14.76 kCal/Mol

miRNA: 3' GA-GU-UGUC-GGGCUUUUGGUAGU 5'

|| :| | || :| |||||||||

SPP1: 5' CTATAGA-AGAAATGCAAACTATCA 3'

**K12-4-3p**

Score: 215

Energy: -14.07 kCal/Mol

miRNA: 3' AGUCG-A-U-CC-G-G-AG-U-C-A-UAAGAU 5'

||| | | || : : || | | | ||||||

SPP1: 5' AAAGCTTCAGGGTTATGTCTATGTTCATTCTA 3'

**PRG1**

**K12-2**

Score: 184

Energy: -19.44 kCal/Mol

miRNA: 3' GUCU-AG-CUGGGCCUGAUG-UCAA 5'

||| || |||::||||||| | ||

PRG1: 5' AAGACTCTGACTTGGATTGTGAATT 3'

**K12-11**

Score: 195

Energy: -15.87 kCal/Mol

miRNA: 3' AGCCUGU-GUCCGAUU-CGU-AAUU 5'

|: |||| ||||| || ||| ||||

PRG1: 5' TT-GACACCAGGC-AATGTAGTTAG 3'

**K12-1**

Score: 209

Energy: -17.85 kCal/Mol

miRNA: 3' CG-AAUGUGGGUCAAAGGACAU-UA 5'

|| |||| |:: ||||||| | ||

PRG1: 5' GCTTTAC-CTTTTTTTCCTGGATAT 3'

**K12-9**

Score: 198

Energy: -17.62 kCal/Mol

miRNA: 3' AAUGCGUC-G-ACG-CA-U-AUGG-GUC 5'

|| ||| : ||| || | |||| ||

PRG1: 5' AAACACAGTTGTGCTGTCAATACCTCAT 3'

**K12-3**

Score: 199

Energy: -15.18 kCal/Mol

miRNA: 3' GCGAC-GGCA-GGA-GUCUU-A-CA-CU 5'

| :|| :|| ::| ||||| | || ||

PRG1: 5' CTTTGAATGTGTTTGCAGAGCTAGTGGA 3'

**S100A2**

**K12-9-3p**

Score: 193

Energy: -19.980000 kCal/Mol

miRNA: 3' UCGCCCCAAAUGCGUCGACC-CA 5'

| | || | :| ||||||| ||

S100A2: 5' A-CCCGGCTGGCTCAGCTGGAGT 3'

**K12-6-5p**

Score: 196

Energy: -16.87 kCal/Mol

miRNA: 3' GG-C-U-A-C-CUAAUCC-ACGAC-GACC 5'

:: | | | | || |||| ||||| ||

S100A2: 5' TTGGAAATCGAGA-TAGGTTGCTGACTTT 3'

Score: 184

Energy: -19.53 kCal/Mol

miRNA: 3' GGCUAC-CUAAUCCAC-GACGACC 5'

|:|: | ||| ||| | || ||||

S100A2: 5' CTGG-GAGATGAGG-GCCTCCTGG 3'

**K12-6-3p**

Score: 187

Energy: -18.19 kCal/Mol

miRNA: 3' GAG-UUGUCG-GG-CUUUU-G-GUA-GU 5'

||| :|| | || ||||| | | | ||

S100A2: 5' CTCTGAC-TCTCCTGGAAATCTC-TCCA 3'

**K12-5**

Score: 198

Energy: -15.32 kCal/Mol

miRNA: 3' G-GCCGUUCAAGGUC-CG-UA-G-GAU 5'

| | |||| |||| || || | |||

S100A2: 5' CTCTCCAAG-GCCAGAGCTATGCTTTA 3'

Score: 168

Energy: -19.68 kCal/Mol

miRNA: 3' GGCCGUUCAAGG-U-CCGUAGGAU 5'

|:|| :|| | | ||| |||||

S100A2: 5' CTGG-GAG-A-TGAGGGCCTCCTG 3'

**K12-4-3p**

Score: 165

Energy: -19.01 kCal/Mol

miRNA: 3' AGUCGAUCCGGAGUC-A-U-AA-GAU 5'

|:||| || ||||| | | | |||

S100A2: 5' CCGGCT-GG-CTCAGCTGGAGTGCTG 3'

**K12-3-5p**

Score: 181

Energy: -19.80 kCal/Mol

miRNA: 3' G-CGA-CG-G-CAGGAGUCUUAC-ACU 5'

| ||| || | | | | |||||| |||

S100A2: 5' CGGCTGGCTCAG-C-T-GGAGTGCTGG 3'

**K12-3-3p**

Score: 163

Energy: -18.70 kCal/Mol

miRNA: 3' AC-AGUGUA-A-G-AC-ACUG-G-CG-C-U 5'

|| || ||| | | |||| | || | |

S100A2: 5' TGTTC-CATAACCCGGCTGGCTCAGCTGGA 3'
