# Supplementary material for: Identification of Cellular Genes Targeted by KSHV-Encoded MicroRNAs
Source: PLoS Pathog. 2007 May 11;3(5):e65. doi: 10.1371/journal.ppat.0030065 (PMC1876501; doi:10.1371/journal.ppat.0030065)
Supplement: Table S1 — (228 KB DOC) [file ppat.0030065.st001.doc]

Supplemental Tables

| |  | **Gene symbol** | **Affymetrix**  **Probe set** | **Fold Change** | **t-value** | **Parametric p-value** | | --- | --- | --- | --- | --- | --- | | **1** | [SOX11](http://www.ncbi.nlm.nih.gov/entrez/query.fcgi?cmd=search&db=gene&term=SOX11) | [204914_s_at](https://www.affymetrix.com/LinkServlet?probeset=204914_s_at) | 1.39 | -8.55 | 0.000141 | | **2** | [CDH11](http://www.ncbi.nlm.nih.gov/entrez/query.fcgi?cmd=search&db=gene&term=CDH11) | [207173_x_at](https://www.affymetrix.com/LinkServlet?probeset=207173_x_at) | 1.36 | -10.33 | 4.80E-05 | | **3** | [ZNF505](http://www.ncbi.nlm.nih.gov/entrez/query.fcgi?cmd=search&db=gene&term=ZNF505) | [208119_s_at](https://www.affymetrix.com/LinkServlet?probeset=208119_s_at) | 1.29 | -8.75 | 0.000123 | | **4** | [GAL](http://www.ncbi.nlm.nih.gov/entrez/query.fcgi?cmd=search&db=gene&term=GAL) | [214240_at](https://www.affymetrix.com/LinkServlet?probeset=214240_at) | 1.24 | -8.52 | 0.000143 | | **5** | [GALNT3](http://www.ncbi.nlm.nih.gov/entrez/query.fcgi?cmd=search&db=gene&term=GALNT3) | [203397_s_at](https://www.affymetrix.com/LinkServlet?probeset=203397_s_at) | 1.14 | -8.92 | 0.000111 | | **6** | [COCH](http://www.ncbi.nlm.nih.gov/entrez/query.fcgi?cmd=search&db=gene&term=COCH) | [205229_s_at](https://www.affymetrix.com/LinkServlet?probeset=205229_s_at) | 1.12 | -11.68 | 2.40E-05 | | **7** | [SLC31A1](http://www.ncbi.nlm.nih.gov/entrez/query.fcgi?cmd=search&db=gene&term=SLC31A1) | [235013_at](https://www.affymetrix.com/LinkServlet?probeset=235013_at) | 1.10 | -9.02 | 0.000104 | | **8** | [AK3](http://www.ncbi.nlm.nih.gov/entrez/query.fcgi?cmd=search&db=gene&term=AK3) | [224655_at](https://www.affymetrix.com/LinkServlet?probeset=224655_at) | 1.05 | -10.83 | 3.70E-05 | | **9** | [SMAP](http://www.ncbi.nlm.nih.gov/entrez/query.fcgi?cmd=search&db=gene&term=SMAP) | [201784_s_at](https://www.affymetrix.com/LinkServlet?probeset=201784_s_at) | -1.05 | 13.48 | 1.00E-05 | | **10** | [CRY1](http://www.ncbi.nlm.nih.gov/entrez/query.fcgi?cmd=search&db=gene&term=CRY1) | [209674_at](https://www.affymetrix.com/LinkServlet?probeset=209674_at) | -1.06 | 9.19 | 9.30E-05 | | **11** | [CDC16](http://www.ncbi.nlm.nih.gov/entrez/query.fcgi?cmd=search&db=gene&term=CDC16) | [202717_s_at](https://www.affymetrix.com/LinkServlet?probeset=202717_s_at) | -1.08 | 8.25 | 0.000172 | | **12** | [DNAJC12](http://www.ncbi.nlm.nih.gov/entrez/query.fcgi?cmd=search&db=gene&term=DNAJC12) | [218976_at](https://www.affymetrix.com/LinkServlet?probeset=218976_at) | -1.08 | 8.15 | 0.000183 | | **13** | [VPS37C](http://www.ncbi.nlm.nih.gov/entrez/query.fcgi?cmd=search&db=gene&term=VPS37C) | [219053_s_at](https://www.affymetrix.com/LinkServlet?probeset=219053_s_at) | -1.10 | 9.21 | 9.20E-05 | | **14** | [SLC1A4](http://www.ncbi.nlm.nih.gov/entrez/query.fcgi?cmd=search&db=gene&term=SLC1A4) | [212811_x_at](https://www.affymetrix.com/LinkServlet?probeset=212811_x_at) | -1.10 | 8.21 | 0.000176 | | **15** | [TBPL1](http://www.ncbi.nlm.nih.gov/entrez/query.fcgi?cmd=search&db=gene&term=TBPL1) | [208398_s_at](https://www.affymetrix.com/LinkServlet?probeset=208398_s_at) | -1.12 | 13.33 | 1.10E-05 | | **16** | [FLJ22639](http://www.ncbi.nlm.nih.gov/entrez/query.fcgi?cmd=search&db=gene&term=FLJ22639) | [220399_at](https://www.affymetrix.com/LinkServlet?probeset=220399_at) | -1.13 | 11.8 | 2.20E-05 | | **17** | [CDC16](http://www.ncbi.nlm.nih.gov/entrez/query.fcgi?cmd=search&db=gene&term=CDC16) | [209658_at](https://www.affymetrix.com/LinkServlet?probeset=209658_at) | -1.13 | 16.82 | 3.00E-06 | | **18** | [FLJ10781](http://www.ncbi.nlm.nih.gov/entrez/query.fcgi?cmd=search&db=gene&term=FLJ10781) | [218824_at](https://www.affymetrix.com/LinkServlet?probeset=218824_at) | -1.14 | 11.06 | 3.30E-05 | | **19** | [TMEPAI](http://www.ncbi.nlm.nih.gov/entrez/query.fcgi?cmd=search&db=gene&term=TMEPAI) | [217875_s_at](https://www.affymetrix.com/LinkServlet?probeset=217875_s_at) | -1.15 | 8.78 | 0.000121 | | **20** | [TPD52](http://www.ncbi.nlm.nih.gov/entrez/query.fcgi?cmd=search&db=gene&term=TPD52) | [201689_s_at](https://www.affymetrix.com/LinkServlet?probeset=201689_s_at) | -1.17 | 10.41 | 4.60E-05 | | **21** | [FGF13](http://www.ncbi.nlm.nih.gov/entrez/query.fcgi?cmd=search&db=gene&term=FGF13) | [205110_s_at](https://www.affymetrix.com/LinkServlet?probeset=205110_s_at) | -1.17 | 9.03 | 0.000103 | | **22** | [FHL2](http://www.ncbi.nlm.nih.gov/entrez/query.fcgi?cmd=search&db=gene&term=FHL2) | [202949_s_at](https://www.affymetrix.com/LinkServlet?probeset=202949_s_at) | -1.18 | 10.04 | 5.70E-05 | | **23** | [TPD52](http://www.ncbi.nlm.nih.gov/entrez/query.fcgi?cmd=search&db=gene&term=TPD52) | [201690_s_at](https://www.affymetrix.com/LinkServlet?probeset=201690_s_at) | -1.18 | 13.75 | 9.00E-06 | | **24** | [TMEM46](http://www.ncbi.nlm.nih.gov/entrez/query.fcgi?cmd=search&db=gene&term=TMEM46) | [230493_at](https://www.affymetrix.com/LinkServlet?probeset=230493_at) | -1.18 | 8.21 | 0.000176 | | **25** | [CXXC5](http://www.ncbi.nlm.nih.gov/entrez/query.fcgi?cmd=search&db=gene&term=CXXC5) | [233955_x_at](https://www.affymetrix.com/LinkServlet?probeset=233955_x_at) | -1.21 | 8.84 | 0.000116 | | **26** | [NXPH4](http://www.ncbi.nlm.nih.gov/entrez/query.fcgi?cmd=search&db=gene&term=NXPH4) | [221967_at](https://www.affymetrix.com/LinkServlet?probeset=221967_at) | -1.21 | 9.46 | 7.90E-05 | | **27** | [LDOC1](http://www.ncbi.nlm.nih.gov/entrez/query.fcgi?cmd=search&db=gene&term=LDOC1) | [204454_at](https://www.affymetrix.com/LinkServlet?probeset=204454_at) | -1.22 | 13.6 | 1.00E-05 | | **28** | [SLC44A5](http://www.ncbi.nlm.nih.gov/entrez/query.fcgi?cmd=search&db=gene&term=SLC44A5) | [235763_at](https://www.affymetrix.com/LinkServlet?probeset=235763_at) | -1.24 | 9.5 | 7.80E-05 | | **29** | [ANXA4](http://www.ncbi.nlm.nih.gov/entrez/query.fcgi?cmd=search&db=gene&term=ANXA4) | [201301_s_at](https://www.affymetrix.com/LinkServlet?probeset=201301_s_at) | -1.26 | 9.17 | 9.40E-05 | | **30** | [RAB27A](http://www.ncbi.nlm.nih.gov/entrez/query.fcgi?cmd=search&db=gene&term=RAB27A) | [209515_s_at](https://www.affymetrix.com/LinkServlet?probeset=209515_s_at) | -1.32 | 10.48 | 4.40E-05 | | **31** | [IL13RA1](http://www.ncbi.nlm.nih.gov/entrez/query.fcgi?cmd=search&db=gene&term=IL13RA1) | [201887_at](https://www.affymetrix.com/LinkServlet?probeset=201887_at) | -1.32 | 14.36 | 7.00E-06 | | **32** | [STAT3](http://www.ncbi.nlm.nih.gov/entrez/query.fcgi?cmd=search&db=gene&term=STAT3) | [208991_at](https://www.affymetrix.com/LinkServlet?probeset=208991_at) | -1.34 | 9.65 | 7.10E-05 | | **33** | [LOC286434](http://www.ncbi.nlm.nih.gov/entrez/query.fcgi?cmd=search&db=gene&term=LOC286434) | [59433_at](https://www.affymetrix.com/LinkServlet?probeset=59433_at) | -1.34 | 10.14 | 5.40E-05 | | **34** | [RAB27A](http://www.ncbi.nlm.nih.gov/entrez/query.fcgi?cmd=search&db=gene&term=RAB27A) | [209514_s_at](https://www.affymetrix.com/LinkServlet?probeset=209514_s_at) | -1.35 | 9.34 | 8.60E-05 | | **35** | [MAOA](http://www.ncbi.nlm.nih.gov/entrez/query.fcgi?cmd=search&db=gene&term=MAOA) | [212741_at](https://www.affymetrix.com/LinkServlet?probeset=212741_at) | -1.38 | 10.78 | 3.80E-05 | | **36** | [EPAS1](http://www.ncbi.nlm.nih.gov/entrez/query.fcgi?cmd=search&db=gene&term=EPAS1) | [200878_at](https://www.affymetrix.com/LinkServlet?probeset=200878_at) | -1.40 | 8.58 | 0.000138 | | **37** | [SLC6A15](http://www.ncbi.nlm.nih.gov/entrez/query.fcgi?cmd=search&db=gene&term=SLC6A15) | [206376_at](https://www.affymetrix.com/LinkServlet?probeset=206376_at) | -1.41 | 11.11 | 3.20E-05 | | **38** | [STAT1](http://www.ncbi.nlm.nih.gov/entrez/query.fcgi?cmd=search&db=gene&term=STAT1) | [209969_s_at](https://www.affymetrix.com/LinkServlet?probeset=209969_s_at) | -1.42 | 11.24 | 3.00E-05 | | **39** | [DKK3](http://www.ncbi.nlm.nih.gov/entrez/query.fcgi?cmd=search&db=gene&term=DKK3) | [214247_s_at](https://www.affymetrix.com/LinkServlet?probeset=214247_s_at) | -1.43 | 9.47 | 7.90E-05 | | **40** | [TACSTD1](http://www.ncbi.nlm.nih.gov/entrez/query.fcgi?cmd=search&db=gene&term=TACSTD1) | [201839_s_at](https://www.affymetrix.com/LinkServlet?probeset=201839_s_at) | -1.45 | 8.81 | 0.000119 | | **41** | [IRS1](http://www.ncbi.nlm.nih.gov/entrez/query.fcgi?cmd=search&db=gene&term=IRS1) | [204686_at](https://www.affymetrix.com/LinkServlet?probeset=204686_at) | -1.46 | 8.53 | 0.000143 | | **42** | [C18orf1](http://www.ncbi.nlm.nih.gov/entrez/query.fcgi?cmd=search&db=gene&term=C18orf1) | [207996_s_at](https://www.affymetrix.com/LinkServlet?probeset=207996_s_at) | -1.46 | 8.61 | 0.000135 | | **43** | [HDHD1A](http://www.ncbi.nlm.nih.gov/entrez/query.fcgi?cmd=search&db=gene&term=HDHD1A) | [203974_at](https://www.affymetrix.com/LinkServlet?probeset=203974_at) | -1.48 | 9.16 | 9.50E-05 | | **44** | [PACRG](http://www.ncbi.nlm.nih.gov/entrez/query.fcgi?cmd=search&db=gene&term=PACRG) | [234096_at](https://www.affymetrix.com/LinkServlet?probeset=234096_at) | -1.49 | 9.64 | 7.20E-05 | | **45** | [UCP2](http://www.ncbi.nlm.nih.gov/entrez/query.fcgi?cmd=search&db=gene&term=UCP2) | [208998_at](https://www.affymetrix.com/LinkServlet?probeset=208998_at) | -1.54 | 15.62 | 4.00E-06 | | **46** | [FN1](http://www.ncbi.nlm.nih.gov/entrez/query.fcgi?cmd=search&db=gene&term=FN1) | [210495_x_at](https://www.affymetrix.com/LinkServlet?probeset=210495_x_at) | -1.54 | 8.76 | 0.000122 | | **47** | [CD44](http://www.ncbi.nlm.nih.gov/entrez/query.fcgi?cmd=search&db=gene&term=CD44) | [212063_at](https://www.affymetrix.com/LinkServlet?probeset=212063_at) | -1.54 | 8.32 | 0.000164 | | **48** | [FKBP11](http://www.ncbi.nlm.nih.gov/entrez/query.fcgi?cmd=search&db=gene&term=FKBP11) | [219117_s_at](https://www.affymetrix.com/LinkServlet?probeset=219117_s_at) | -1.56 | 9.06 | 0.000101 | | **49** | [FN1](http://www.ncbi.nlm.nih.gov/entrez/query.fcgi?cmd=search&db=gene&term=FN1) | [212464_s_at](https://www.affymetrix.com/LinkServlet?probeset=212464_s_at) | -1.59 | 9.23 | 9.10E-05 | | **50** | [MAGEH1](http://www.ncbi.nlm.nih.gov/entrez/query.fcgi?cmd=search&db=gene&term=MAGEH1) | [218573_at](https://www.affymetrix.com/LinkServlet?probeset=218573_at) | -1.60 | 14.23 | 8.00E-06 | | **51** | [FN1](http://www.ncbi.nlm.nih.gov/entrez/query.fcgi?cmd=search&db=gene&term=FN1) | [211719_x_at](https://www.affymetrix.com/LinkServlet?probeset=211719_x_at) | -1.61 | 8.26 | 0.000171 | | **52** | [HSPA1A](http://www.ncbi.nlm.nih.gov/entrez/query.fcgi?cmd=search&db=gene&term=HSPA1A) | [200799_at](https://www.affymetrix.com/LinkServlet?probeset=200799_at) | -1.65 | 10.1 | 5.50E-05 | | **53** | [C10orf58](http://www.ncbi.nlm.nih.gov/entrez/query.fcgi?cmd=search&db=gene&term=C10orf58) | [228155_at](https://www.affymetrix.com/LinkServlet?probeset=228155_at) | -1.65 | 8.71 | 0.000127 | | **54** | [FZD10](http://www.ncbi.nlm.nih.gov/entrez/query.fcgi?cmd=search&db=gene&term=FZD10) | [219764_at](https://www.affymetrix.com/LinkServlet?probeset=219764_at) | -1.67 | 9.1 | 9.90E-05 | | **55** | [MAP2K5](http://www.ncbi.nlm.nih.gov/entrez/query.fcgi?cmd=search&db=gene&term=MAP2K5) | [211371_at](https://www.affymetrix.com/LinkServlet?probeset=211371_at) | -1.74 | 9.57 | 7.40E-05 | | **56** | [AKR1C3](http://www.ncbi.nlm.nih.gov/entrez/query.fcgi?cmd=search&db=gene&term=AKR1C3) | [209160_at](https://www.affymetrix.com/LinkServlet?probeset=209160_at) | -1.76 | 12 | 2.00E-05 | | **57** | [LZTS1](http://www.ncbi.nlm.nih.gov/entrez/query.fcgi?cmd=search&db=gene&term=LZTS1) | [47550_at](https://www.affymetrix.com/LinkServlet?probeset=47550_at) | -1.82 | 8.92 | 0.000111 | | **58** | [ABCA1](http://www.ncbi.nlm.nih.gov/entrez/query.fcgi?cmd=search&db=gene&term=ABCA1) | [203505_at](https://www.affymetrix.com/LinkServlet?probeset=203505_at) | -1.87 | 9.33 | 8.60E-05 | | **59** | [HTATIP2](http://www.ncbi.nlm.nih.gov/entrez/query.fcgi?cmd=search&db=gene&term=HTATIP2) | [209448_at](https://www.affymetrix.com/LinkServlet?probeset=209448_at) | -1.88 | 15.32 | 5.00E-06 | | **60** | [INHBA](http://www.ncbi.nlm.nih.gov/entrez/query.fcgi?cmd=search&db=gene&term=INHBA) | [227140_at](https://www.affymetrix.com/LinkServlet?probeset=227140_at) | -1.92 | 17 | 3.00E-06 | | **61** | [UCHL1](http://www.ncbi.nlm.nih.gov/entrez/query.fcgi?cmd=search&db=gene&term=UCHL1) | [201387_s_at](https://www.affymetrix.com/LinkServlet?probeset=201387_s_at) | -1.93 | 29.61 | p < 0.000001 | | **62** | [SPINT2](http://www.ncbi.nlm.nih.gov/entrez/query.fcgi?cmd=search&db=gene&term=SPINT2) | [210715_s_at](https://www.affymetrix.com/LinkServlet?probeset=210715_s_at) | -1.99 | 10.25 | 5.00E-05 | | **63** | [BHLHB5](http://www.ncbi.nlm.nih.gov/entrez/query.fcgi?cmd=search&db=gene&term=BHLHB5) | [228636_at](https://www.affymetrix.com/LinkServlet?probeset=228636_at) | -2.02 | 12.64 | 1.50E-05 | | **64** | [PRG1](http://www.ncbi.nlm.nih.gov/entrez/query.fcgi?cmd=search&db=gene&term=PRG1) | [201858_s_at](https://www.affymetrix.com/LinkServlet?probeset=201858_s_at) | -2.03 | 10.71 | 3.90E-05 | | **65** | [ARMCX6](http://www.ncbi.nlm.nih.gov/entrez/query.fcgi?cmd=search&db=gene&term=ARMCX6) | [214749_s_at](https://www.affymetrix.com/LinkServlet?probeset=214749_s_at) | -2.09 | 19.42 | 1.00E-06 | | **66** | [ADFP](http://www.ncbi.nlm.nih.gov/entrez/query.fcgi?cmd=search&db=gene&term=ADFP) | [209122_at](https://www.affymetrix.com/LinkServlet?probeset=209122_at) | -2.20 | 16.86 | 3.00E-06 | | **67** | [SPOCK3](http://www.ncbi.nlm.nih.gov/entrez/query.fcgi?cmd=search&db=gene&term=SPOCK3) | [235342_at](https://www.affymetrix.com/LinkServlet?probeset=235342_at) | -2.26 | 8.25 | 0.000172 | | **68** | [IFI16](http://www.ncbi.nlm.nih.gov/entrez/query.fcgi?cmd=search&db=gene&term=IFI16) | [208966_x_at](https://www.affymetrix.com/LinkServlet?probeset=208966_x_at) | -2.35 | 11.32 | 2.80E-05 | | **69** | [IFI16](http://www.ncbi.nlm.nih.gov/entrez/query.fcgi?cmd=search&db=gene&term=IFI16) | [206332_s_at](https://www.affymetrix.com/LinkServlet?probeset=206332_s_at) | -2.45 | 13.03 | 1.30E-05 | | **70** | [IFI16](http://www.ncbi.nlm.nih.gov/entrez/query.fcgi?cmd=search&db=gene&term=IFI16) | [208965_s_at](https://www.affymetrix.com/LinkServlet?probeset=208965_s_at) | -2.73 | 11.85 | 2.20E-05 | | **71** | [PLAC8](http://www.ncbi.nlm.nih.gov/entrez/query.fcgi?cmd=search&db=gene&term=PLAC8) | [219014_at](https://www.affymetrix.com/LinkServlet?probeset=219014_at) | -2.81 | 8.29 | 0.000167 | | **72** | [PDZK1](http://www.ncbi.nlm.nih.gov/entrez/query.fcgi?cmd=search&db=gene&term=PDZK1) | [205380_at](https://www.affymetrix.com/LinkServlet?probeset=205380_at) | -2.92 | 8.81 | 0.000118 | | **73** | [IF](http://www.ncbi.nlm.nih.gov/entrez/query.fcgi?cmd=search&db=gene&term=IF) | [1555564_a_at](https://www.affymetrix.com/LinkServlet?probeset=1555564_a_at) | -3.24 | 11.22 | 3.00E-05 | | **74** | [ITM2A](http://www.ncbi.nlm.nih.gov/entrez/query.fcgi?cmd=search&db=gene&term=ITM2A) | [202746_at](https://www.affymetrix.com/LinkServlet?probeset=202746_at) | -4.06 | 8.21 | 0.000176 | | **75** | [PRG1](http://www.ncbi.nlm.nih.gov/entrez/query.fcgi?cmd=search&db=gene&term=PRG1) | [201859_at](https://www.affymetrix.com/LinkServlet?probeset=201859_at) | -4.27 | 26.44 | p < 0.000001 | | **76** | [IF](http://www.ncbi.nlm.nih.gov/entrez/query.fcgi?cmd=search&db=gene&term=IF) | [203854_at](https://www.affymetrix.com/LinkServlet?probeset=203854_at) | -4.44 | 12.03 | 2.00E-05 | | **77** | [S100A2](http://www.ncbi.nlm.nih.gov/entrez/query.fcgi?cmd=search&db=gene&term=S100A2) | [204268_at](https://www.affymetrix.com/LinkServlet?probeset=204268_at) | -5.03 | 18.04 | 2.00E-06 | | **78** | [MID1IP1](http://www.ncbi.nlm.nih.gov/entrez/query.fcgi?cmd=search&db=gene&term=MID1IP1) | [218251_at](https://www.affymetrix.com/LinkServlet?probeset=218251_at) | -5.53 | 28.41 | p < 0.000001 | | **79** | [THBS1](http://www.ncbi.nlm.nih.gov/entrez/query.fcgi?cmd=search&db=gene&term=THBS1) | [201110_s_at](https://www.affymetrix.com/LinkServlet?probeset=201110_s_at) | -6.10 | 11.07 | 3.20E-05 | | **80** | [EST](http://www.ncbi.nlm.nih.gov/entrez/query.fcgi?cmd=search&db=gene&term=) | [244567_at](https://www.affymetrix.com/LinkServlet?probeset=244567_at) | -9.02 | 13.96 | 8.00E-06 | | **81** | [SPP1](http://www.ncbi.nlm.nih.gov/entrez/query.fcgi?cmd=search&db=gene&term=SPP1) | 209875_s_at | -20.44 | 22.77 | p < 0.000001 | |
| --- | --- | --- | --- | --- | --- | --- | --- | --- | --- | --- | --- | --- | --- | --- | --- | --- | --- | --- | --- | --- | --- | --- | --- | --- | --- | --- | --- | --- | --- | --- | --- | --- | --- | --- | --- | --- | --- | --- | --- | --- | --- | --- | --- | --- | --- | --- | --- | --- | --- | --- | --- | --- | --- | --- | --- | --- | --- | --- | --- | --- | --- | --- | --- | --- | --- | --- | --- | --- | --- | --- | --- | --- | --- | --- | --- | --- | --- | --- | --- | --- | --- | --- | --- | --- | --- | --- | --- | --- | --- | --- | --- | --- | --- | --- | --- | --- | --- | --- | --- | --- | --- | --- | --- | --- | --- | --- | --- | --- | --- | --- | --- | --- | --- | --- | --- | --- | --- | --- | --- | --- | --- | --- | --- | --- | --- | --- | --- | --- | --- | --- | --- | --- | --- | --- | --- | --- | --- | --- | --- | --- | --- | --- | --- | --- | --- | --- | --- | --- | --- | --- | --- | --- | --- | --- | --- | --- | --- | --- | --- | --- | --- | --- | --- | --- | --- | --- | --- | --- | --- | --- | --- | --- | --- | --- | --- | --- | --- | --- | --- | --- | --- | --- | --- | --- | --- | --- | --- | --- | --- | --- | --- | --- | --- | --- | --- | --- | --- | --- | --- | --- | --- | --- | --- | --- | --- | --- | --- | --- | --- | --- | --- | --- | --- | --- | --- | --- | --- | --- | --- | --- | --- | --- | --- | --- | --- | --- | --- | --- | --- | --- | --- | --- | --- | --- | --- | --- | --- | --- | --- | --- | --- | --- | --- | --- | --- | --- | --- | --- | --- | --- | --- | --- | --- | --- | --- | --- | --- | --- | --- | --- | --- | --- | --- | --- | --- | --- | --- | --- | --- | --- | --- | --- | --- | --- | --- | --- | --- | --- | --- | --- | --- | --- | --- | --- | --- | --- | --- | --- | --- | --- | --- | --- | --- | --- | --- | --- | --- | --- | --- | --- | --- | --- | --- | --- | --- | --- | --- | --- | --- | --- | --- | --- | --- | --- | --- | --- | --- | --- | --- | --- | --- | --- | --- | --- | --- | --- | --- | --- | --- | --- | --- | --- | --- | --- | --- | --- | --- | --- | --- | --- | --- | --- | --- | --- | --- | --- | --- | --- | --- | --- | --- | --- | --- | --- | --- | --- | --- | --- | --- | --- | --- | --- | --- | --- | --- | --- | --- | --- | --- | --- | --- | --- | --- | --- | --- | --- | --- | --- | --- | --- | --- | --- | --- | --- | --- | --- | --- | --- | --- | --- | --- | --- | --- | --- | --- | --- | --- | --- | --- | --- | --- | --- | --- | --- | --- | --- | --- | --- | --- | --- | --- | --- | --- | --- | --- | --- | --- | --- | --- | --- | --- | --- | --- | --- | --- | --- | --- | --- | --- | --- | --- | --- | --- | --- | --- | --- | --- | --- | --- | --- | --- | --- | --- | --- | --- | --- | --- | --- | --- | --- | --- | --- | --- | --- | --- | --- | --- | --- | --- | --- | --- | --- | --- | --- | --- | --- | --- | --- | --- | --- | --- | --- | --- | --- | --- | --- | --- | --- | --- | --- | --- | --- | --- | --- | --- | --- | --- | --- | --- | --- | --- | --- |

**Table S1**. List of changed genes with 100% cross validation.
