# Supplementary material for: Identification of Cellular Genes Targeted by KSHV-Encoded MicroRNAs
Source: PLoS Pathog. 2007 May 11;3(5):e65. doi: 10.1371/journal.ppat.0030065 (PMC1876501; doi:10.1371/journal.ppat.0030065)
Supplement: Table S2 — (56 KB DOC) [file ppat.0030065.st002.doc]

| **construct** | **primer 1** | **position** | **primer 2** | **position** |
| --- | --- | --- | --- | --- |
| **miRNA cluster** | TCCCAGTAGAGTGACCCAG | 119099 | GTACGCGGTTGTTTACGCAG | 121966 |
|  |  |  |  |  |
| **miRNA expresion** | |  |  |  |
| **miRK-12-1** | AATCTGGTTGACGGACTTTC | 121783 | GTACGCGGTTGTTTACGCAG | 121947 |
| **miRK-12-3** | GTTCGTCGCTTGGACCTGGAG | 121522 | GTCCCCAAACTCCCAACCAA | 121639 |
| **miRK-12-4** | GAGGTTTGAGAGGCGTAGACATCC | 121364 | CTCCAGGTCCAAGCGACGAAC | 121522 |
| **miRK-12-5** | CCCGCATAGGTTTTTGTGG | 121216 | GGATGTCTACGCCTCTCAAACCTC | 121364 |
| **miRK-12-6** | ACACAGAACAATAACGGGCGACTA | 120727 | TAAAGCGGGCGTTCGTAAGC | 120861 |
| **miRK-12-11** | AAAAATTGCCGCCGTGAAGGTC | 120520 | TTCATCATTTCACCCACCGTCTCT | 120699 |
| **miRK-12-7** | CTACACTAAGCCCGAACG | 120293 | CGTGCCCACCGATGAGATAC | 120446 |
| **miRK-12-8** | TAGCAGGGCCATCCACAC | 119884 | TGACAAAGCATGCACTGGAAATC | 120031 |
| **miRK-12-9** | TGCTTCCGGAAATACCACCTGAGT | 119220 | TGAGTCATCGCAGCCCCTATTC | 119384 |
|  |  |  |  |  |
| **3'UTR Luc.** |  |  |  |  |
| **THBS1** | TCTAGATGAAAGACTGATCATAAACCAATGC | | GGCCGGCCTGTGTACAAAAAAAAGCACATTCCT | |
| **SPP1** | TCTAGATGCTTCTTTCTCAGTTTATTGGTTG | | GGCCGGCCTTAATTGCTGGACAACCGTG | |
| **PRG1** | TCTAGATTGACACCAGGCAATGTAGT | | GGCCGGCCACCTTTGGTTATATTTCTGC | |
| **HS6ST2** | GGGATATCTGGCTCAAAAAGGCCTGTACATACT | | GGGATATCGGTTTGTAAAGCTACACCAATGGAC | |
|  |  |  |  |  |
| **miRNA sensor** |  |  |  |  |
| **miRK-12-1** | CTAGACTTACACCCAGTTTCCTGTAATCTCGAGCTTACACCCAGTTTCCTGTAATGGCCGG | | | |
| **miRK-12-3-5p** | CTAGATCGCTGCCGTCCTCAGAATGTGACTCGAGTCGCTGCCGTCCTCAGAATGTGAGGCCGG | | | |
| **miRK-12-4-3p** | CTAGATCAGCTAGGCCTCAGTATTCTACTCGAGTCAGCTAGGCCTCAGTATTCTAGGCCGG | | | |
| **miRK-12-5** | ACCGGCAAGTTCCAGGCATCCTATTATTACCGGCAAGTTCCAGGCATCCTAGGCCGG | | | |
| **miRK-12-6-3p** | CTAGACTCAACAGCCCGAAAACCATCACTCGAGCTCAACAGCCCGAAAACCATCAGGCCGG | | | |
| **miRK-12-11** | CTAGAATCGGACACAGGCTAAGCATTAATTATTATCGGACACAGGCTAAGCATTAAGGCCGG | | | |
| **miRK-12-7** | CTAGAGAGCGCCAGCAACATGGGATCACTCGAGGAGCGCCAGCAACATGGGATCAGGCCGG | | | |
| **miRK-12-8** | CTAGAGTGCTCTCTCAGTCGCGCCTAGCTCGAGGTGCTCTCTCAGTCGCGCCTAGGGCCGG | | | |
| **miRK-12-9-3p** | CTAGAACGCAGCTGCGTATACCCAGCCTCGAGACGCAGCTGCGTATACCCAGCGGCCGG | | | |
|  |  |  |  |  |
| **qRT-PCR** |  |  |  |  |
| **transcript** | **primer 1** |  | **primer 2** |  |
| RAB27A | TCGTAGCTTAACGACAGCGTTCTT | | TGCGAGTGCTATGGCTTCCT |  |
| PRG1 | ATGATGCAGAAGCTACTCAAATGC | | AGTCCTGGATTCTCGTCTTTGG |  |
| SPP1 | CTGTGGCCACATGGCTAAACC |  | GGTGAGACTCATCAGACTGGTGA |  |
| ITM2A | CAGAACTCAGATACTGACCGGCA | | GAAGGGAATTTGCAGGATCCTC |  |
| S100A2 | ACCTGGTCTGCCACAGATCCAT |  | TTCTCATCCAGGCTGCCCAT |  |
| THBS1 | ATCCAAAGCGTCTTCACCAGAGA |  | TGGCCAATGTAGTTAGTGCGG |  |
| B-actin | AAATCTGGCACCACACCTTC |  | TCCATCACGATGCCAGTGGT |  |

**Table S2.** List of primers for the cloning of miRNA expression and –sensor constructs.
